# Living with pathological narcissism: core conflictual relational themes within intimate relationships

**DOI:** 10.1186/s12888-021-03660-x

**Published:** 2022-01-10

**Authors:** Nicholas J. S. Day, Michelle L. Townsend, Brin F. S. Grenyer

**Affiliations:** Illawarra Health and Medical Research Institute and School of Psychology, University of Wollongong, Wollongong, Australia

## Abstract

**Background:**

Pathological narcissism is a severe mental health condition that includes disturbances in interpersonal functioning. Interpersonal difficulties by those affected include aggressive, domineering, cold and coercive behaviours which often result in strong negative reactions from others. We sought to examine the moment-to-moment patterns that emerge within close relationships between intimate partners and family members.

**Methods:**

Participants (*N* = 15) were romantic partners (73.3%) and family members (26.6%) in a close and long-term relationship (+ 10 years) with an individual with pathological narcissism. Participants told verbatim relationship narratives involving five narrative interactions with their relative with pathological narcissism and five narrative interactions with others. Transcripts were coded using the using Core Conflictual Relationship Theme method. Participants also completed three versions of the Relationship Questionnaire, reporting on 1. their relationship style ‘in general’, 2. their relationship style ‘with their relative’ and 3. the relationship style of their relative.

**Results:**

A total of 133 relationship episodes were analysed, comprising 783 components (wishes, responses of others and responses of self). While the identified wishes (e.g., for love, for support) were consistent between relative and non-relative narratives, there was significantly higher disharmony and lower harmony in narratives involving relatives with pathological narcissism. Described disharmony in these relationships involved the relative’s rejecting, subjugating and attacking behaviours, and participants rejecting and withdrawing behaviours. There was a prominent deactivation of participants attachment system when interacting with their relative with pathological narcissism, endorsing predominately dismissing relationship styles. Individuals with pathological narcissism were similarly rated as predominately dismissing.

**Conclusions:**

Together, these results reflect the cycles of interpersonal dysfunction for individuals with pathological narcissism and their partners and family members. Treatment implications point to the risk of therapists withdrawing and dismissing a patient with high pathological narcissism in the countertransference. Strategies to monitor and manage these core relational themes in treatment remain a challenge.

## Introduction

Interpersonal dysfunction is a well-documented aspect of pathological narcissism [1–4]. Indeed, a number of the criteria for narcissistic personality disorder as they appear in the *Diagnostic and Statistical Manual of Mental Disorders* (DSM-5, [5]) infer or overtly state an impairment of interpersonal relationships (e.g. “Is interpersonally exploitative, i.e., takes advantage of others to achieve his or her own ends” (criteria [6], p. 670). Similarly, the alternate model of personality disorders specifies the instrumental function of interpersonal relationships towards self-esteem (identity and self-direction criteria) and impaired quality of relationships, which may present as a lack of empathy, superficiality and trait antagonism for individuals with narcissistic personality disorder.

One avenue for understanding interpersonal dysfunction for individuals with pathological narcissism has been in the treatment context, given documented difficulties in establishing an effective therapeutic alliance with patients with narcissistic preoccupations [6, 7]. The concept of ‘transference’ was described by Freud [8] as “a whole series of [revived] psychological experiences … not as belonging to the past, but as applying to the person of the physician of the present moment” (p. 116). In the treatment of patients with narcissistic personalities, patterns of transference and countertransference can be particularly intense [9, 10], as “dysfunctional modes of relatedness are inevitably recreated in the treatment context” ([11], p. 185). Corresponding countertransference from clinicians have been documented, such as feeling a difficulty connecting, feeling excluded, becoming overly solicitous, becoming aggressive and competitive, feeling idealized and grandiose, feeling scrutinised and engaging in mutual admiration [12]. When activated, the reconciliation of such intense transference and countertransference patterns have been identified as crucial for effective therapeutic work [13, 14], however outside of therapy such relationship patterns are the cause of significant pain and distress to others [15].

This study aims to extend this research by investigating the “dysfunctional modes of relatedness” ([11], p. 185) of individuals with pathological narcissism through the relationship patterns described by partners and family members. One method of exploring an individual’s relationship patterns is via the Core Conflictual Relationship Theme (CCRT; [16]), in which individuals describe specific relationship narratives. The CCRT explores not only an individual’s characteristic way of interacting with others, but also their fantasised or longed for outcomes of interactions, and has been used to understand the dysfunctional relationship patterns of individuals with personality disorders [17–19]. For instance Bourke and Grenyer [20], utilising the CCRT, describe the disharmonious relationship patterns of mutual disengagement and withdrawal between therapists and patients with borderline personality disorder [20], potentially linked to therapists intense emotional reactions to such patients [21]. Such research highlights a complex intersubjective dynamic at play [22], whereby pathological intrapersonal processes appear as both the cause of – and simultaneously in response to – negative interpersonal perceptions and interactions with others [17, 23, 24].

Indeed, recent research on pathological narcissism highlights the complex interactions between perceptions of self and other, and related affective processes with corresponding shifts in mentalizing modes or defensively split object relations [25]. For instance, narcissistic features were found to be associated with both perceptions of others as cold whilst acting cold towards others [26], associated with both acting aggressively towards other and receiving aggression from others [27], and to perceive others as dominant and respond with negative emotionality and antagonism [28]. Understanding such dysfunctional interpersonal patterns and perceptions is crucial, as it not only helps identify and contain destructive enactments within the therapy [29], but also for fostering positive relationship patterns for both individuals with pathological narcissism and their partners and family members.

### Study Aims

This study seeks to understand patterns of interpersonal functioning for individuals with pathological narcissism and their partners and family members. For this research, partners and family members will be referred to as ‘participants’, individuals with pathological narcissism will be referred to as the ‘relative’ and others will be described as ‘non-relatives’.

Given the documented interpersonal dysfunction identified for individuals with pathological narcissism [1, 3] and the intense countertransference reported by clinicians treating individuals with NPD [11, 30], it is predicted that relationship narratives with individuals with pathological narcissism will have significantly higher incidence of disharmony and lower levels of harmony than other relationship narratives. Regarding relationship style, it has been suggested that dismissing attachment is the prototypical organisation for individuals with narcissistic personality disorder [31]. Further, as being in a relationship with individuals with pathological narcissism may inspire feelings of dependency, insecurity and vulnerability [32], it is expected that individuals with pathological narcissism will be described as displaying a dismissing relationship style and that participants will report insecure relationship styles in general. However, it is also expected that participants will report greater insecurity in their relationship style when interacting with their relative with pathological narcissism.

## Methods

### Recruitment

Participants were partners and family members in a close relationship with an individual with pathologically narcissistic traits. All participants provided written informed consent for their responses to be used in research, following institutional review board approval. Participants that had taken part in previous research ([e.g. [33]) were separately invited to participate in the current study. These participants were recruited through invitations posted on various mental health websites that provide information and support that is narcissism specific (e.g. ‘Narcissistic Family Support Group’). Recruitment was advertised as being specifically in relation to a relative with narcissistic traits. Presence of pathologically narcissistic traits were screened through completing an informant version of a brief pathological narcissism inventory (described in measures section).

### Participants

Inclusion criteria were: (1) Participants stating having a “close personal relationship” with a relative with pathological narcissism. (2) Relatives being rated as displaying prominent features of pathological narcissism, adopting a cut off of 36 (average 3) on a narcissism screening measure (SB-PNI-CV). (3) Participants narratives being of sufficient length ([> 70 words, [34]) and receiving an adequate completeness of narrative rating ([> 2.5, [16]) for purposes of analysis. (4) Participants completing measures and demographic information as part of the survey. The sample consisted of 15 participants, achieving a redundancy in themes and sufficient saturation for analysis, reflecting a sample size similar to other studies analysing qualitative responses [35, 36] and comparative with other studies utilizing the CCRT [37].

Table 1 outlines the demographic information of participants and the relative included in the study. All participants stated they had been in a relationship with their relative with pathological narcissism for over 10 years, 40% (*n* = 6) of participants stated their relative has received a formal diagnosis of a mental health condition, a subsample of which included a diagnosis of a personality disorder (26.7%, *n* = 4).

**Table 1:** Demographics for participants (partners and family) and their relatives (people high in pathological narcissism) (*N* = 15)

		Participants (*n* = 15)	Relative (*n* = 15)	
Mean age in years (SD)		52.7 (12.6)	54.9 (11.5)	
Gender				
	Male	6.7% (*n* = 1)	73.3% (*n* = 11)	
	Female	93.3% (*n* = 14)	26.7% (*n* = 4)	
Employment				
	Full time	54.5% (*n* = 6)	54.5% (*n* = 6)	
	Part time	27.3% (*n* = 3)	18.2% (*n* = 2)	
	Unemployed	18.2% (*n* = 2)	27.3% (*n* = 3)	
Relationship				
	Spouse/partner	33.3% (*n* = 5)		
	Former spouse/partner	40% (*n* = 6)		
	Family – Mother	13.3% (*n* = 2)		
	Family – Sibling	13.3% (*n* = 2)		
Is your relationship still current?				
	Yes	46.7% (n = 7)		
	No	53.3% (n = 8)		

### Measures

*Pathological Narcissism Inventory (Carer Version) (SB-PNI-CV).* Schoenleber, Roche, et al. [38] developed a short version of the Pathological Narcissism Inventory (SB-PNI; super brief) as a 12-item measure consisting of the 12 best performing items for the Grandiosity and Vulnerability composites (6 of each) of the Pathological Narcissism Inventory [39]. This measure was then adapted into a carer version (SB-PNI-CV) in the current research, consistent with previous methodology [15] by changing all self-referential terms (i.e. ‘I’) to refer to the relative (i.e. ‘my relative’). The scale operates on a Likert scale from 0 (‘not at all like my relative’) to 5 (‘very much like my relative’) in which higher scores indicate the presence of pathologically narcissistic traits. Informant-based methods of investigating narcissism and its effects have previously been found provide meaningful perspectives on clinical phenomenon not captured in self-report methods [40, 41]. The SB-PNI-CV demonstrated acceptable internal consistency (α = 0.75). This measure utilised a cut off score of 36 (average score of 3) consistent with previous research ([e.g., [15]), requiring included participants to, on average, endorse the presence of narcissistic pathology in their relative.

*Core Conflictual Relationship Theme—Leipzig/Ulm* ([CCRT-LU, [16, 42]). The CCRT-LU is an established method for understanding and formulating an individual’s central relationship patterns. Luborsky [43] developed the Relational Anecdote Paradigm (RAP) for a research setting and involves participants describing events in relationships that include specific interactions with specific people. Participants were given a textbox to respond to the prompt in as much detail as they would like. Participants were asked to provide 10 narratives in total (five involving their relative with pathological narcissism, five involving someone who is not their relative). Participants were presented with the following text, specified as either relative or non-relative narratives:

“Tell us of five incidents or events, each involving yourself and your relative. Each one should be a specific incident. Some should be current and some old incidents. For each one tell (1) where it occurred, (2) some of what your relative said or did (3) some of what you said or did, (4) what happened at the end, and (5) when the event happened. They can be any incident you want, it just has to be about a specific event that was personally important or a problem to you in some way.”

Analysis of relationship narratives involves the identification of specific units as they appear, classified as wishes (W), response of other (RO) and response of self (RS). Each scorable unit is then coded according to the Leipzig/Ulm hierarchical categories (reflecting dichotomous harmonious and disharmonious interactions) when forming an individual’s CCRT-LU profile [42]. All codable units were included in analysis. For example, the text “My friend and I had dinner and my relative phoned me non-stop during dinner so that I had to keep excusing myself. I was embarrassed and did not attempt to visit with friends after that” contains elements such as a ‘wish’ (to enjoy time with a friend, code: ‘C. Loving, Feeling Well’), a disharmonious ‘response of other’ (being pressured and interrupted by relative, code: ‘K. Subjugating’) and disharmonious ‘response of self’ (feeling embarrassed, avoiding friend, codes: ‘F. Being Dissatisfied’, ‘M. Withdrawing’).

The CCRT-LU has demonstrated reliability and validity for a range of psychological disorders [17, 18, 37, 44–46]. Inter-rater reliability for CCRT-LU coding was completed on 10% of data by a second, independent and trained rater. Overall inter-rater reliability was calculated at *k* = *0*.78, consisting of reliability for coding presence (agreement of relevant sections of text for coding, *k* = 0.72) and coding agreement (raters coding the same interactions within harmonious and disharmonious clusters, *k* = 0.84). This score reflects a very good consensus between independent raters [47].

*Relationship Questionnaire* ([RQ, [48]). The RQ is a 4-item questionnaire designed to measure adult relationship styles across the dimensions: secure, pre-occupied, dismissing and fearful. Participants respond on a 7-point Likert scale (1 = not at all like me; 7 = very much like me), which was then transformed into a score between 0 – 100 to facilitate ease of interpretation consistent with prior literature [49]. In order to increase specificity and generalizability of the RQ results, an adapted version was used in which participants respond 1. in general relationships, 2. in specific relationships and 3. providing a rating of their relative. Informant versions of the RQ have been validated in empirical research [50]. The scale has demonstrated convergent validity with other measures of attachment and structured interviews, correctly classifying 92% of cases [48]. Evidence for the reliability and stability of the RQ have been demonstrated [51] as well as cross-cultural validation [52].

Statistical analyses.

Statistical testing was used to compare mean differences in participants scores, for example, when comparing identified instances of ‘harmonious’ and ‘disharmonious’ interactions in participant narratives involving a relative with pathological narcissism. To do this, all identified interactions were classified into dichotomous groups (harmonious or disharmonious) and were summed across the five narratives provided by each participant. This resulted in each participant having a single score for either ‘harmonious’ or ‘disharmonious’ interactions, which was combined and averaged across all participants, allowing for a comparison of mean scores. As mean scores used for comparison were generated from the same 15 participants (i.e., both CCRT and RQ comparisons) paired samples t-test were used throughout to assess for significance in observed differences. A significance level of 0.05 was selected for statistical tests.

## Results

Table 2 displays the mean scores of the narcissism measure, as well as the mean continuous scores of the RQ.

**Table 2:** Descriptive statistics of participant scores (*n* = 15) for measures under examination

Measure	Subfactor	Mean (SD)
Pathological Narcissism Inventory (Carer Version)		3.7 (0.8)
	Grandiose	4 (0.9)
	Vulnerable	3.4 (1)
Relationship Questionnaire (of Self)		
	Secure	47.6 (31.7)
	Fearful	48.6 (36.5)
	Pre-occupied	33.3 (32.2)
	Dismissing	58.1 (29.3)
Relationship Questionnaire (of Relative)		
	Secure	18.1 (24.4)
	Fearful	44.8 (38.5)
	Pre-occupied	29.5 (34.3)
	Dismissing	54.3 (43.3)
Relationship Questionnaire (of Self with Relative)		
	Secure	2.9 (5.9)
	Fearful	57.1 (40)
	Pre-occupied	28.6 (41.8)
	Dismissing	75.2 (39.1)

### Relationship Narratives

A total of 133 relationship narratives were described by participants, with a total of 783 individual components identified within participant narratives comprising either wishes (W, *n* = 118), response of other (RO, *n* = 358) and response of self (RS, *n* = 307) categories.

### Wishes

**Fig. 1:**
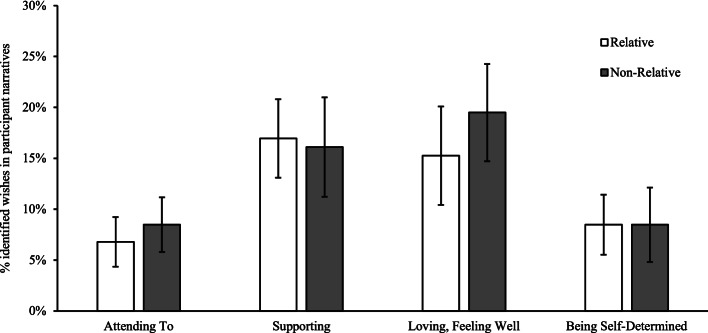
Percentage of wishes as described by participants. *Note*. Error bars indicate standard error

A total of 118 wishes were coded from participant narratives. There were no significant differences in wishes between relative and non-relative narratives. However, regardless of relationship type (relative or non-relative), participants consistently indicated significantly higher wishes for love and support (*M* = 5.3, *SD* = 3.5), compared to other wishes (*M* = 2.5, *SD* = 2.4), *t*(14) = -4.4, *p* = 0.001. Percentage of wishes identified within participant narratives are displayed in Fig. 1.

### Interpersonal Dysfunction

**Fig. 2:**
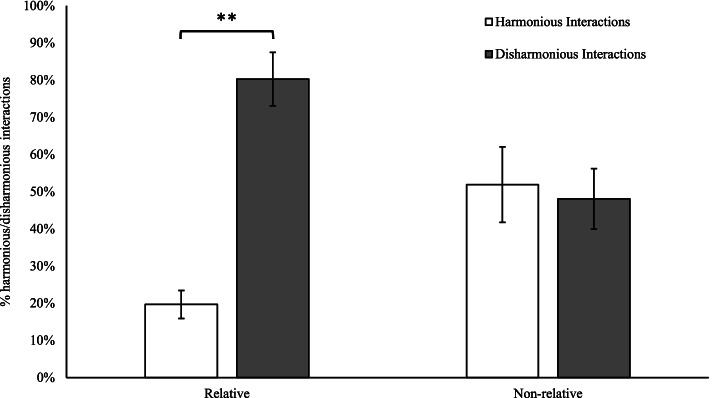
Percentage of harmonious and disharmonious interactions between relatives with pathological narcissism and non-relatives. *Note*. Error bars indicate standard error. **significant at α < .01

Participant narratives involving non-relatives contained approximately equivalent harmonious (*M* = 10.9, *SD* = 8.3) and disharmonious (*M* = 10.1, *SD* = 6.6) interactions. Conversely, narratives involving a relative with pathological narcissism involved significantly lower harmony (*M* = 4.5, *SD* = 3.3) and elevated disharmony (*M* = 18.2, *SD* = 6.3), *t* (14) = 8.48, *p* = 0.001. Percentage of harmonious and disharmonious interactions between relatives and non-relatives are presented in Fig. 2.

**Fig. 3:**
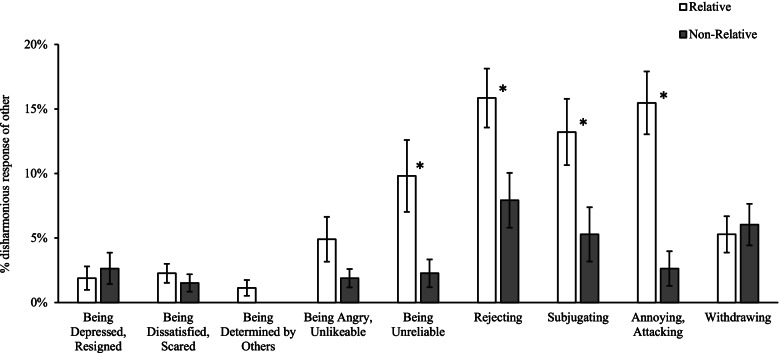
Percentage disharmonious RO’s as described by participants. *Note*. Error bars indicate standard error. *significant at α < .05

Direction of disharmony was further investigated as either *from* relatives/non-relatives (i.e., response of other, RO) or *towards* relatives/non-relatives (i.e., response of self, RS). Figure 3 displays disharmonious RO’s, which include elevated instances of rejecting, subjugating, annoying, attacking, and unreliable responses from individuals with pathological narcissism.

**Fig. 4:**
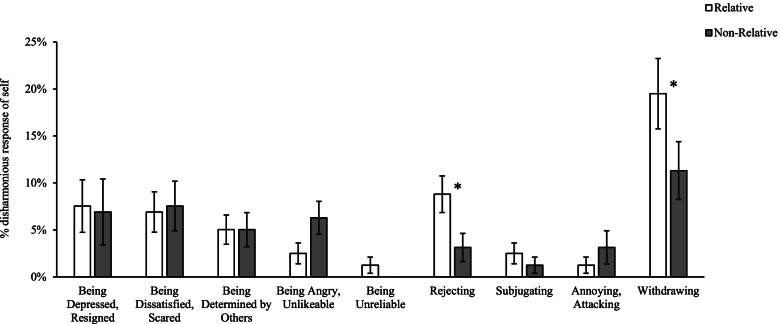
Percentage disharmonious RS’s as described by participants. *Note*. Error bars indicate standard error. *significant at α < .05

Figure 4 displays disharmonious RS’s, which include elevated instances of participants rejecting and withdrawing behaviour towards relatives with pathological narcissism.

### Relationship Styles

**Fig. 5:**
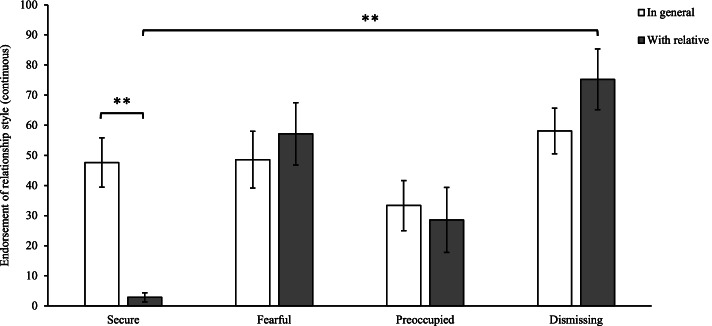
Change in self-report relationship style ‘in general’, compared to when interacting with relative with pathological narcissism. *Note.* Error bars indicate standard error. RQ scores have been linear transformed between 0 – 100. **significant at α < .01

Continuous scores of participant relationship style ‘in general’ found no significant differences between relationship styles. Comparing participants scores ‘in general’ and when interacting ‘with [their] relative’ with pathological narcissism, participants reported a significantly decreased ‘secure’ relationship style *t* (14) = 5.36, *p* = 0.001. When interacting with their relative, participants insecure relationship styles were all significantly greater than ‘secure’ relationship style, with the highest scores corresponding with the ‘dismissing’ relationship style *t* (14) = -5.44, p = 0.001. These scores are displayed in Fig. 5.

**Fig. 6:**
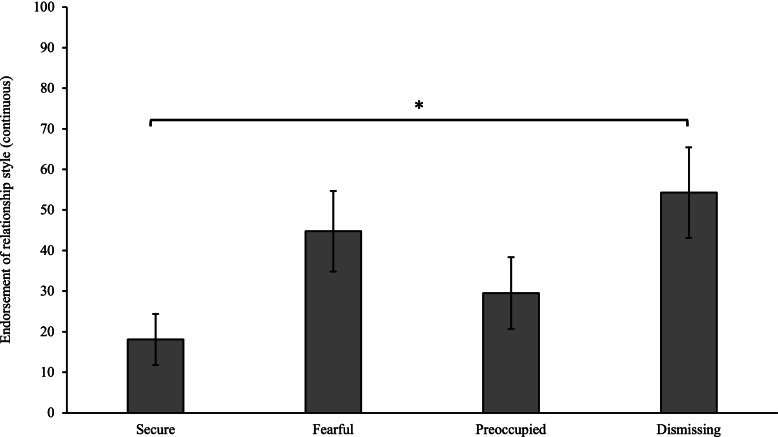
Informant report of relationship style of relatives with pathological narcissism. *Note.* Error bars indicate standard error. *Note*. Error bars indicate standard error. RQ scores have been linear transformed between 0 – 100. *significant at α < .05

Participants also completed an informant version of the RQ, reporting on the perceived style of their relative with pathological narcissism. Continuous ratings indicated a significant difference between relatives ‘dismissing’ and ‘secure’ scores *t* (14) = -2.6, *p* = 0.019. These results are displayed in Fig. 6.

## Discussion

This study examined the described interpersonal style and patterns of interaction between an individual with pathological narcissism and informant participants. While relationship wishes (e.g., for love, support) were not significantly different between groups, narratives with relatives with pathological narcissism had significantly greater disharmony, involving instances of relatives attacking, rejecting and subjugating behaviours, and participants rejecting and withdrawing behaviours. Overall, narratives with non-relatives typically involved equal instances of harmony and disharmony, where relationship conflicts were satisfactorily resolved, and relationship wishes were fulfilled. In contrast, narratives with relatives with pathological narcissism involved escalating relationship conflicts, whereby both participants and relatives became increasingly conflictually entrenched and disconnected, and relationship wishes remained unfulfilled. Further, when interacting with their relative with pathological narcissism, participants rated their relationship style to be significantly less secure, and more dismissive, and similarly rated their relative as predominately dismissive.

These results provide meaningful examples of interpersonal patterns whereby both participants and relatives became locked in dysfunctional modes of relatedness. Interestingly, the prevalence of wishes was not significantly different between relative and non-relative narratives. This is perhaps not surprising as early writings and findings regarding CCRT elements found that wishes are stable across relationships as “apparently, one’s wishes, needs and intentions in relationships are relatively intractable” ([p. 160, [53]). This finding strengthens the confidence in the results, as the relationships could not be viewed as having fundamentally different motivations between relatives and non-relatives, but rather suggests a unique pathological interpersonal process that occurs within relative narratives that disrupts functioning. These findings do suggest, however, that participants in this sample were particularly motivated by interpersonal wishes for love and support in their interpersonal relationships, perhaps indicating primacy of dependency rather than autonomy. This is consistent with previous research, suggesting that those in relationships with individuals with pathological narcissism may be particularly fragile and vulnerable to interpersonal exploitation [32].

Typically, narratives involving individuals with pathological narcissism were more concrete, included non-mentalising descriptions of behaviour, and ended with unresolved relationship ruptures. For example,

“While visiting my relative at his home, he made several insults to my appearance including my weight, hair style and colour, and clothing. I walked away. He then insulted my children and used several inappropriate racial epithets towards them. I got my family up and we left.” (Participant #24).

This is in contrast with narratives involving non-relatives, which were typically more reflective, involving consideration of the others mind and perspective, and in which relationship ruptures were reconciled in mutually satisfying ways. For example,

“I have a co-worker whom I respect greatly. We were co-teaching, but she had been out of town for some time. During this time, I had run the class by myself and had gotten in the mental habit of thinking it was my class. When she got back, she said she felt that I had put her in the role of being an assistant instead of a co-teacher. I reviewed things I said to the students, and I realized she was right. I apologized to her and made sure we had equal responsibility from then on.” (Participant #9).

Interpersonal dysfunction is known to be a highly prevalent feature of pathological narcissism [54], involving vindictive, domineering and cold interpersonal styles [2, 3, 55, 56]. However recent research has highlighted the complex dynamics that inform such dysfunctional interpersonal processes [25]. Involving, for instance, individuals with pathological narcissism perceiving others as more dominant, cold or aggressive, and thereby respond in similar ways [24, 26–28]. The results of the current study highlight that it may not only a perception of others that inform pathological interpersonal processes, but that in reality individuals become more withdrawn, dismissive and rejecting towards individuals with pathological narcissism. It is important to note, however, that we are not suggesting that participants are somehow ‘wrong’ or ‘bad’ in responding to their relatives in such withdrawn or rejecting ways, as there may be very necessary reasons for doing so. For instance, research has indicated individuals with pathological narcissism to exhibit emotional, sexual, physical and verbal abuse towards their partners and family members [32, 57], and indeed narratives shared within this research indicated similar themes. However, this finding does underscore the importance of understanding the way that others interact with and react to individuals with pathological narcissism, in order to understand the way the dysfunctional intrapersonal and interpersonal mechanisms of the disorder are sustained.

Two potential implications of the current research are presented. First, broadly, participants can be described as responding to their relative with a deactivated attachment [58], likely to preserve the integrity of self-functioning and to minimise intense and destabilising affective processes associated with such relationships [15]. In this, it is interesting that participants reported relationship style became less secure and more similar to their relatives when interacting with them. As research and theoretical accounts have indicated the defensive nature of narcissistic grandiosity, providing a façade of self-stability in an attempt to regulate potentially overwhelming affects [59, 60], it may be that when interacting with their relative, participants relational style begins to mirror that of their relative for similar purposes. Second, these findings have crucial implications regarding the psychological treatment of narcissistic pathology. Research reports that common therapist countertransference towards individuals with pathological narcissism involves feelings of anger, disengagement and inadequacy [10, 11]. These findings highlight the possibility that patients with pathological narcissism may replicate patterns of interpersonal dysfunction within the therapeutic relationship, involving instances of dismissiveness and antagonism towards clinicians ([e.g., [61, 62]). As such, in such instances it is important for treating clinicians to not ‘enact’ reciprocal dysfunctional behaviours [22], involving a defensive withdrawal, deactivation of attachment systems and engagement of non-mentalising modes. But rather, therapists attempt to explore with the patient the co-created atmosphere of disengagement, and attempt to facilitate the generation of insight through the process of rupture and repair.

### Limitations

A number of limitations should be considered in the interpretation of this study. First, while the included sample is adequate for CCRT methodology [37] as well as for qualitative analyses more broadly [35, 36], it is still relatively small. As such, a standalone interpretation of quantitative results such as the RQ may require caution, and should be viewed as supporting information regarding the qualitative results presented, which involved over 500 coded relationship elements. Second, the use of only a brief informant narcissism measure may limit the ability to infer conclusions regarding the narcissism construct in this study. While informant reporting of personality pathology, and pathological narcissism specifically, has been demonstrated to provide meaningful and valid clinical information [41, 62, 63], relatives in this study did not necessarily have a specific diagnosis of narcissistic personality disorder. In other words, this sample reports on individuals with ‘pathological narcissism’ reflecting a broad umbrella term that includes diverse narcissistic presentations [63], and establishes that the narcissism features displayed were pathological and deleterious to interpersonal relationships. Third, the use of retrospective participant narratives to explore the interpersonal patterns of individuals with pathological narcissism and their partners and family is associated with both advantages and disadvantages. While one key advantage of this approach is the degree of depth that can be explored through the analysis of moment-to-moment interactions as described within narratives. One key disadvantage is the degree of participant bias that may impact the accuracy of events contained in described narratives, as participants may be motivated to provide narratives that overemphasise the negative quality of their relative with pathological narcissism. A more ‘objective’ approach may be the direct observation of dyad interactions between participants and relatives within a clinical or research setting, which would lessen the influence and impact of bias on the research team’s interpretation of interaction patterns. Such an approach is a suggested avenue for future research. That being said, a further advantage of the use of retrospective narratives in this study is the inclusion of everyday ‘real world’ interaction examples for analysis, that may not spontaneously arise under the scrutiny of a laboratory setting. Indeed, in some ways the accuracy or ‘truth’ contained in participant narratives is less important than the subjective representation that such narratives provide, as subjective representations of self and others are what guide human interaction and are implicated in the conflictual patterns that emerge. Finally, while the CCRT has a robust clinical and research history demonstrating its reliability and validity [37, 64], there are limitations to the CCRT methodology that are relevant for interpreting the results of the present study. While all components of the CCRT (W, RO, RS) require some level of inference by the researcher in order to identify and categorise interactions, this is particularly true for the ‘wish’ component which may vary anywhere from wishes being literally expressed (e.g., “I want him to…”) to being more abstractly presented [65]. As such, a number of precautions were taken to limit the over-interpretation of wishes (and indeed other CCRT components) from extending beyond the text as provided by participants. This included the use of consensus categories [42] which help to restrict the identification of CCRT components to specific examples that are more concrete readily identifiable. Similarly, the use of a second-inter rater with experience in CCRT methodology helps strengthen the confidence in findings as a high degree of reliability was identified between raters. That being said, while it is not a standard component of CCRT methodology, future research designs may include directly asking participants about their ‘wish’ in relation to their narrative (i.e., “what did you want out of, or hope for, in this situation with your relative?”) in order to examine this dimension of the CCRT more fully.

## Conclusion

Kealy and Ogrodniczuk [66] outline the “obstruction of love” for individuals with pathological narcissism, which includes both love of others, and paradoxically, love of self. In this way, individuals with pathological narcissism struggle with healthy self-regulation and positive self-regard [67], as the inflated and grandiose self is fragile, unable to tolerate the normal experience of human fallibility, and rather necessitates a constant rigid view of the self as exceptional [60]. As such, interpersonal relationships for individuals with pathological narcissism serve two, contradictory functions. First, they serve to bolster the grandiose self through identification with idealised, perfected others. Second, they serve as a platform to evacuate all negative projections of the self onto devalued, rejected others. This cycle, repeated with employers, friends, family and romantic partners, typifies the tragedy of intimate relationships for individuals with pathological narcissism in that “they are unable to elicit the responses from others that will stabilize their self-esteem that they so desperately long for” [12, p. 208]. Indeed, this is reflected in the findings of our sample, as participants did not become more solicitous, caring and attentive when interacting with their relative with pathological narcissism—they became more rejecting, withdrawing and dismissing. As such, our results demonstrate a dynamic of interpersonal dysfunction between participants and their relatives that are likely both in response to, and sustain the, disorder of pathological narcissism. Treatment implications include therapists attending to patterns of transference and countertransference in the therapeutic alliance that may mirror patterns of interpersonal dysfunction within the patients’ wider relationships, including instances of mutual dismissal, rejection and withdrawal.

## Data Availability

The datasets generated during and/or analysed during the current study are not publicly available due to the sensitive and personal nature of participant qualitative responses but are available from the corresponding author on reasonable request.
